# Investigation into the prognostic factors of early recurrence and progression in previously untreated diffuse large B-cell lymphoma and a statistical prediction model for POD12

**DOI:** 10.3389/fimmu.2025.1539924

**Published:** 2025-08-06

**Authors:** Ke Lian, Wenyao Zhu, Zhihui Hu, Fang Su, CaiXia Xu, Hui Wang

**Affiliations:** ^1^ Department Shanxi Bethune Hospital, Shanxi Academy of Medical Sciences, Tongji Shanxi Hospital, Third Hospital of Shanxi Medical University, Taiyuan, China; ^2^ Department of Radiotherapy, The Affiliated Yantai Yuhuangding Hospital of Qingdao University, Yantai, China; ^3^ Department of Cancer Prevention and Control, Shanxi Province Cancer Hospital Shanxi Hospital Affiliated to Cancer Hospital, Chinese Academy of Medical Sciences Cancer Hospital Affiliated to Shanxi Medical University, Taiyuan, China; ^4^ Department of Oncology, Houma People’s Hospital, Houma, Shanxi, China; ^5^ School of Data Sciences, Zhejiang University of Finance and Economics, Hangzhou, China

**Keywords:** diffuse large B cell lymphoma, prognosis, prediction model of POD12, early progression, risk factor

## Abstract

**Objective:**

The objective of this study is to evaluate the incidence, prognostic value, and risk factors of progression of disease within 12 months (POD12) in patients with diffuse large B-cell lymphoma (DLBCL).

**Methods:**

A retrospective analysis of the clinical, pathological, and follow-up data was carried out on 69 DLBCL cases in Shanxi Bethune Hospital from January 2016 to June 2020. One-way ANOVA and multivariate Cox regression analysis were used to explore the correlation between POD12 and prognosis, and logistic regression analysis was used to explore the risk factors of POD12, accompanied by prediction models based on convolutional neural networks and long short-term memory (CNN-LSTM), as well as particle swarm optimization and general regression neural network (PSO-GRNN) models.

**Results:**

(1) POD12 is significantly correlated with PFS (*p<* 0.001) and OS (*p* = 0.008). (2) From the univariate logistic regression analysis corrected by the first-line chemotherapy regimen, LDH, β_2_-MG, stage, ECOG, NLR, and SII are identified as risk factors for POD12 (*p<* 0.1), while β_2_-MG and ECOG are identified as independent risk factors from the multivariate logistic regression analysis (*p*< 0.05). (3) A prediction model for POD12 is established based on LDH, β_2_-MG, stage, ECOG, NLR, and SII. The AUC is 0.846 (95% CI: 0.749~0.944, *p<* 0.001), suggesting that the model is reasonable. A prediction method for the characteristic variables of POD12 risk is proposed using the CNN-LSTM deep learning model based on chaotic time series. Comparatively, the CNN-LSTM and PSO-GRNN models are the most suitable to predict the risk level of the POD12 in the future.

## 1 Introduction

Diffuse large B-cell lymphoma (DLBCL) is a type of hematological malignancy with high heterogeneity in biological characteristics, clinical manifestations, and prognosis, which is influenced by many factors. Although more than 60% of patients can achieve a complete cure or long-term survival by the first-line standard immunochemotherapy, a large number of patients still eventually die due to disease recurrence or treatment resistance (1, 2).

Many recent investigations (3, 4) have found that, among DLBCL patients receiving first-line antitumor therapy, disease progression within 12 months after diagnosis (POD12) is a significant adverse prognostic factor, and these patients may not even benefit from rescue treatment combined with autologous hematopoietic stem cell transplantation (AHSCT). Therefore, it is necessary to provide personalized therapy beyond standard immunochemotherapy based on the first-line treatment to improve patient survival, and it is crucial to identify such patients effectively at an early stage.

In addition, since POD12 can only be evaluated after treatment, it cannot be used to guide decision-making in previously untreated patients, which limits its clinical value. Therefore, how to predict POD12 is an urgent problem to be solved. However, at present, there are few investigations on the predictive factors of POD12 (4), and thus, no effective prediction tools or methods for POD12 are available in the clinic.

The objective of this study is to evaluate the prognostic values and risk factors of POD12 through cohort analysis, and to further establish a clinical prediction model of POD12 to provide a theoretical basis for accurate prognostic stratification and personalized therapy for DLBCL.

## 2 Materials and methods

### 2.1 Case data

A retrospective analysis was carried out on 69 DLBCL cases, of which 6 were lost and 63 were complete, in Shanxi Bethune Hospital from January 2016 to June 2020. Inclusion criteria: DLBCL diagnosed and graded by the latest diagnostic standards of the World Health Organization (WHO) in 2008, and first-line chemotherapy, and complete medical records and follow-up data. Exclusion criteria: (1) Double-hit or triple-hit patients confirmed by the fluorescence *in situ* hybridization (FISH) detection in the pathological tissues; (2) Patients with other malignant tumors; (3) Those who lost follow-up or died without POD12 after diagnosis. The intact DLBCL paraffin tissue preserved in the pathology department was collected and consecutively sectioned into 4μm thickness for immunohistochemical detection. The clinical data of patients were collected, including the sexual distinction, age, clinical stage, ECOG score, absolute value of neutrophils and lymphocytes, platelet and hemoglobin levels, etc. The values of the neutrophil to lymphocyte ratio (NLR), platelet-to-lymphocyte ratio (PLR), lymphocyte to monocyte ratio (LMR), and systemic immune-inflammatory index (SII) were calculated, and the corresponding optimal cutoff values were obtained according to the ROC curve.

### 2.2 Methods

#### 2.2.1 Immunohistochemistry

The antibodies, such as Myc, Bcl-2, Bcl-6, CD10, MUM-1, Ki-67, and P53, were all purchased from Beijing Zhongshan Jinqiao Biotechnology Co. Ltd. (Beijing, China) and the secondary antibody reagents were obtained from Ventana Medical Systems (Rotkreuz, Switzerland). The EnVision two-step method was employed using the Roche BenchMark XT automatic immunohistochemical instrument. Myc protein was localized in the nucleus and, based on the percentage of positive cells, was graded as follows: grade 0 (0), grade 1 (1%~25%), grade 2 (26%~50%), grade 3 (51%~75%), and grade 4 (76%~100%), respectively (8). Bcl-2 positivity is defined as the presence of tumor cells (≥ 50%) with brownish-yellow particles in the cytoplasm and membrane, while Bcl-6 positivity is defined as the presence of brownish-yellow particles (≥ 50%) in the nuclei (5, 6). When the percentage of tumor cells with the brownish-yellow granules in the cytoplasm or nuclei is more than 30%, the positive expression of CD10 or MUM-1 is confirmed. Based on the number of P53-positive cells in the nuclei, expression is graded as follows: 0 (no positive cells), grade 1 (≤ 5%), grade 2 (6%~10%), grade 3 (11%~40%), grade 4 (≥ 41%), respectively, and grades 2~4 are considered the overexpression (7). In addition, the immunohistochemical results of Myc, Bcl-2, CD10, Bcl-6, and MUM-1 proteins were analyzed using the Hans method (9), and the germinal center B (GCB) and non-GCB subtypes were determined according to the COO types.

#### 2.2.2 FISH method

The tumor area was selected based on HE staining, using C-Myc, Bcl-2, and Bcl-6 gene break-apart probes purchased from Guangzhou Anbiping Pharmaceutical Technology Co. Ltd. (Guangzhou, China). More than 100 nuclear signals of tumors were recorded under high-power magnification (× 100) with a Zeiss Axioimager fluorescence microscope. Each probe consists of a red fluorescent and a green fluorescent component. In normal cells, two yellow fusion signals or adjacent red and green signals are observed. In cases of gene translocation, a separation between the yellow with red signals is observed. When the ratio of the separated signal exceeds 10%, the result is considered positive. If more than three yellow fusion signals are present in the same nucleus, it is regarded as a gene duplication.

#### 2.2.3 Follow-up

Telephone and outpatient inquiries were the main follow-up method, and follow-up continued until 31 December 2021. The follow-up period was from the time of diagnosis to the end of follow-up or the date of death. POD12 refers to recurrence or progression within 12 months after diagnosis (3, 4). To accurately observe and compare the effect of POD12 on the subsequent survival of DLBCL patients, the prognostic value of POD12 was verified. The definition of overall survival (OS) in this study is consistent with other relevant studies (3, 4), referring to the interval from 1 year after diagnosis (non-POD12 group) or the date of POD determination (POD group) to the patient’s death or the last follow-up.

### 2.3 Statistical analysis

SPSS 24.0 software was used for the statistical analysis. One-way analysis of variance (ANOVA) and multivariate Cox regression analysis were used to explore the correlation between POD12 and the prognosis of DLBCL. Univariate and multivariate logistic regression analyses were used to explore the risk factors of POD12. The calculation method of model prediction accuracy was defined as the ratio of the total number of true-negative and true-positive patients to the total number of patients. The calculation method of model prediction accuracy is confirmed.

## 3 Deep learning prediction model of CNN-LSTM and PSO-GRNN

### 3.1 Prediction of the POD12 state parameters based on the phase space reconstruction

**Figure 1:**
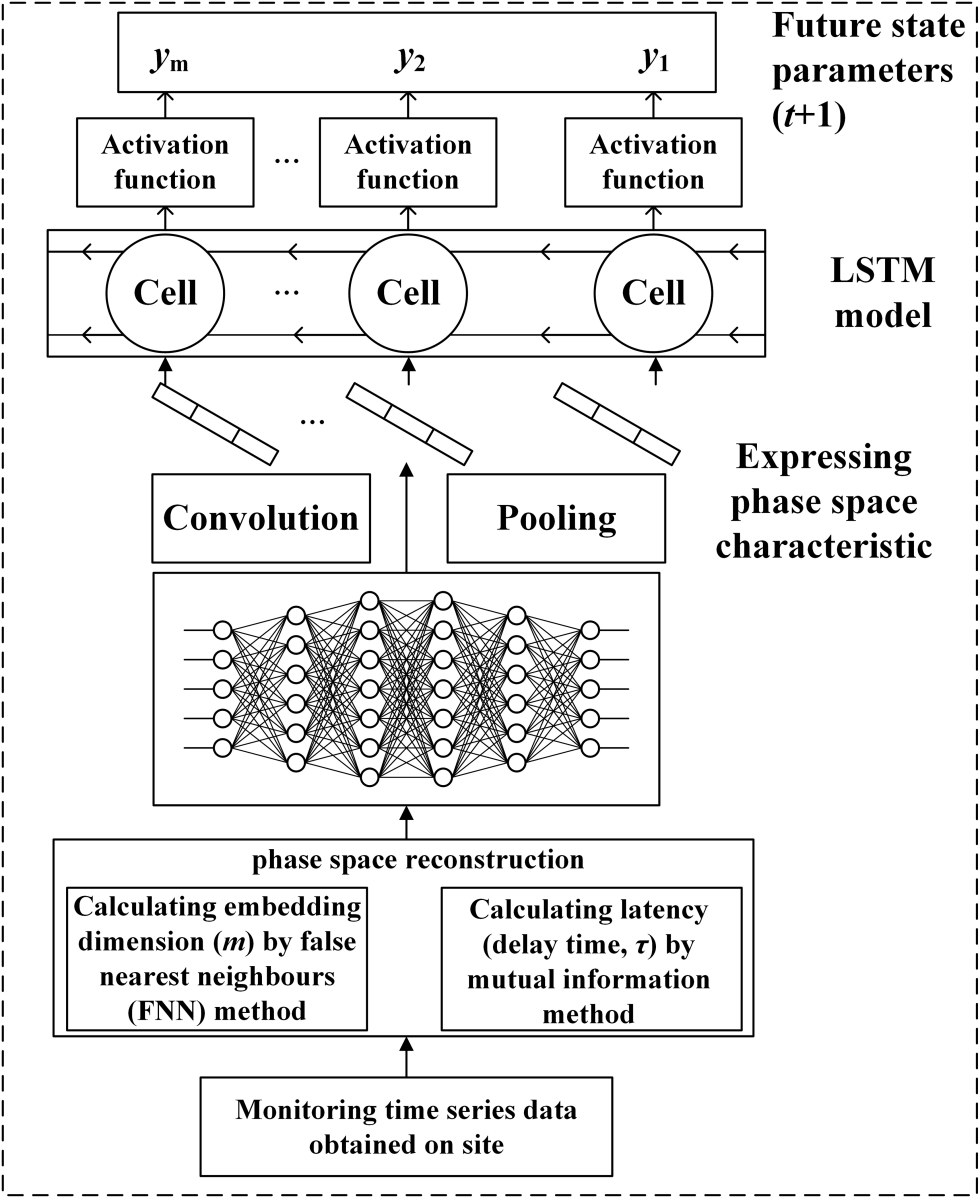
Prediction flowchart of the future state using the CNN-LSTM model.

Aiming at the chaos of the characteristic variables of POD12, the time series data were first reconstructed, and two parameters of the reconstructed phase space were obtained, namely the embedding dimension (*m*), calculated by the false nearest-neighbor (FNN) method, and the latency [delay time (*τ*)], by the mutual information method, to restore the original space. The values of *m* and *τ* were then taken as the input items for the CNN model to extract the sequence of spatial features, and the state of the POD12 characteristic variable at time *t* could be predicted using the LSTM model (see **Figure 1**).

### 3.2 Phase space reconstruction of characteristic variables of POD12

The monitoring time series data exhibit obvious chaotic characteristics. To restore the original space, it is necessary to calculate the embedding dimension (*m*) and latency (*τ*) variables of the phase space for the reconstructed POD12 state. Based on chaos theory, the delay time was calculated using the mutual information method, and the embedding dimension was determined using the false nearest-neighbor method. The phase spaces of the original monitoring data were then reconstructed using these parameters to restore their real spaces. According to the embedding theorem, for the monitoring time-series data ${\left\{X_{i,n}\right\}}_{n=1}^{N}$ (*i* = 1, 2,ċ, *I*; where *I* is the number of characteristic variables and *N* is the length of the time series), the reconstruction space state of the *i*th characteristic variable could be expressed as Equation 1


(1) $$\begin{array}{c} X_{i,n}=\left[X_{i,n},X_{i,n-\tau_{i}}\ldots X_{i,n-\left(m_{i}-1\right)\tau_{i}}\right]=\\ \left[\begin{array}{cccc} x_{i}\left(1\right) & x_{i}\left(2\right) & \cdots & x_{i}\left(n-\left(m_{i}-1\right)\tau_{i}\right)\\ x_{i}\left(1+\tau_{i}\right) & x_{i}\left(2+\tau_{i}\right) & \cdots & x_{i}\left(n-\left(m_{i}-2\right)\tau_{i}\right)\\ \vdots & \vdots & \vdots & \vdots \\ x_{i}\left(1+\left(m_{i}-1\right)\tau_{i}\right) & x_{i}\left(2+\left(m_{i}-1\right)\tau_{i}\right) & \cdots & x_{i}\left(n\right) \end{array}\right] \end{array}$$

Where *X_i,n_* denotes the phase space of the *i*th characteristic variable, and *X_i_* is a point in the phase space of the *i*th characteristic variable. *m_i_* and *τ_i_* are the embedding dimension and delay time of the *i*th characteristic variable, with *n*∈[1, 2, …, *N*]. The dimension number (*m*) of the state space formed by *X_n_* could be expressed as Equation 2:


(2) $$m=\sum_{i=1}^{I}m_{\text{i}}$$

After the phase space reconstruction, there is a mapping function *G*: *R^m^* → *R^m^*, subject to Equation 3


(3) $$X_{n\;+\;l}=G\left(X_{n}\right)$$

Or there is a mapping function *G_i_*: *R^m^* → *R*, subject to Equation 4


(4) $$X_{i,\;n\;+\;l}=G_{i}\left(X_{n}\right)$$

Where *l* denotes the number of the prediction steps. In other words, the state of the time series *X_n + l_* (i.e., the next *l* steps) can be predicted based on the reconstructed state variable.

### 3.3 Prediction of POD12 characteristics based on the CNN-LSTM model

CNN is used to extract the spatial characteristics of the reconstructed phase space. CNN is a model that performs convolution operations within a deep network and has strong expressive ability for spatial data. It is well known that the occurrence of POD12 is not only a gradual process of incubation and evolution but also a nonlinear dynamic process. To further describe this nonlinear process, LSTM is used to predict the characteristic quantity of POD12. LSTM can dynamically memorize historical information and has been widely used. By using LSTM to learn new information from the original POD12 monitoring data while maintaining the status of the historical information of the monitoring data, the new information from the original POD12 monitoring data could be fully learned, and the status of the historical information of the monitoring data could be completely remembered by using LSTM.

**Figure 2:**
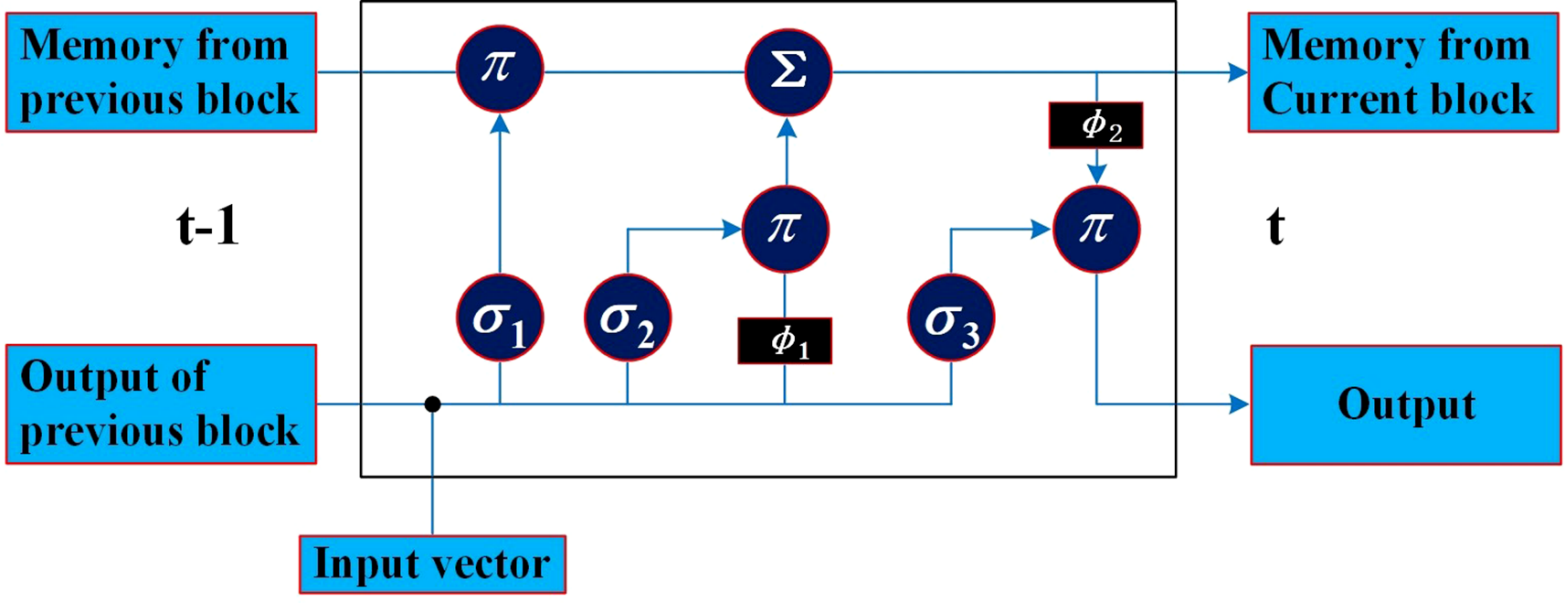
LSTM repeating module.

The LSTM repeated module is composed of three activation function gates (i.e., forget gate (*σ*_1_), input gate (*σ*_2_), and output gate (*σ*_3_)) and two tanh activation functions $\phi_{1}$ $\phi_{2}$ regarding the output, as shown in **Figure 2**. The bullet symbol (·) represents the concatenation operation, and the *π* and Σ symbols represent element-wise multiplication and addition, respectively. The fundamental component of LSTM is the cell state, where a line comes from the previous block memory (*S_t_*_− 1_) and connects to the current block memory (*S_t_*). Afterward, the flow of information straight down the line is allowed. In other words, the forgetting part of the memory in the cell state is determined by the input of the cell state at the previous time point in the forget gate, the memory, and the intermediate output of the cell state. The data to be added to the cell state are adjusted by the sigmoid function, and the intermediate output is determined by the updated memory cell state and the output. In this work, the characteristic sequence of the phase space extracted and reconstructed by CNN was modeled using an LSTM model. By learning the characteristic time series, the nonlinear process of POD12 evolution is represented, the future state of POD12 (i.e., at time *t* + 1) is predicted more accurately, and the risk level of POD12 is evaluated according to the future state of the POD12 characteristic variables.

The CNN-LSTM model consists of the CNN and LSTM parts. The former is used to express and extract the spatial feature information of the reconstructed phase space as the input of LSTM, while the latter is used to receive the output of the CNN-extracted information and use its memory characteristics in the time series to accurately extract time-series features. Thus, the future POD12 state could be predicted. The main process of predicting the future state variables of the POD12 characteristics with the CNN-LSTM model is as follows.

(1) In the CNN part, the reconstructed phase space state represented by a two-dimensional matrix $X_{i,n}=\left[X_{i,n},X_{i,n-\tau_{i}},\cdot \cdot \cdot ,X_{i,n-(m_{i}-1)\tau_{i}}\right]$ is taken as the input. In this matrix, the “row” represents the reconstructed phase space point with a length of *m*, and the “column” represents the length of the time series. This two-dimensional matrix is input to the CNN, and the high-dimensional characteristic information is then extracted using the convolution function *H*(*x*), as shown in Equation 5. After the important characteristics are selected through the pooling layer, the input is transformed into a one-dimensional vector by the flatten layer, and thus, the time series with high-dimensional characteristic information is output.


(5) $$H\left(x\right)=f⊗g=\int_{-\infty }^{+\infty }f\left(x-u\right)\cdot g\left(u\right)$$

Where *f* and *g* are integrable functions, and *x* and *u* are the variables within them.

(2) In the LSTM part, the output time series from the CNN is used as the input. A traditional recurrent neural network (RNN) is prone to gradient disappearance or explosion when handling long sequences. To overcome these problems caused by long-term memory, a gating mechanism is introduced into LSTM, which greatly improves the ability of the data expression for RNN. In general, the characteristic time series are predicted using a single-layer structure in LSTM. During model training, unreasonable training data often lead to overfitting. The *L*_2_ parametric regularization method (weight loss) can be used to mitigate this to some extent. In this method, a regularization item is added to the objective function to bring it closer to the origin by attenuating the weight. The weight loss function of the optimization objective can be expressed as Equation 6:


(6) $$\text{loss}\left(y,W\right)={∥y-\hat{y}∥}_{2}+\lambda {∥W∥}_{2}$$

Where *λ* is the hyperparameter and *W* is the hyperlink weight of the neural network.

(3) CNN-LSTM model training. Firstly, the dataset is divided into training and test sets. Next, the hyperparameters (i.e., epoch data and loss functions) and the optimizer are set, including parameters such as weight and learning rate. Finally, the LSTM model is trained by using the training data to extract the temporal features of the reconstructed phase space data.

(4) After the CNN-LSTM model is successfully trained, the test set is input to obtain the prediction results.

(5) The accuracy of the prediction results in step (4) is evaluated. If the accuracy meets the predefined threshold, step (6) is proceeded; otherwise, step (3) is returned to, and training is continued until the model converges.

(6) The evolution state value of the POD12 at time *t* + 1 is predicted by using the above model trained perfectly.

The prediction process of the risk level for POD12 includes two steps. (1) Quantitative discrimination of the risk level of POD12—that is, the discrimination of the risk level of POD12 at the time of *t* + 1. Firstly, the future state values of the characteristic variables obtained by the CNN-LSTM model (i.e., the state values of the POD12 characteristic variables at the time of *t* + 1) are taken as the input. Based on the PSO-GRNN model, the risk level of POD12 at the time of *t* + 1 is then obtained by the regression method. (2) Prediction of the risk level of POD12 in the future. To predict the POD12 risk level of the test set based on the monitoring variables, the test set data are used as input variables to obtain the future state value of the POD12 risk level of the test set. At the same time, this serves as a comparative experiment to verify the effectiveness of the method in predicting the future risk level of POD12.

GRNN is an artificial neural network that uses the radial basis function as its activation function. Compared with the backpropagation (BP) neural network, it has stronger approximation and learning capabilities. Even with a small sample size, it can effectively identify the risk index of POD12. The purpose of introducing the PSO algorithm is to obtain a more stable regression prediction model. If the smoothing factor of the GRNN model is selected manually, issues such as low efficiency and reduced accuracy may arise. The determination of the smoothing factor is essentially an optimization problem—that is, by finding an optimal smoothing factor, the mean square deviation between the output values and the actual values of the training sample is minimized. By this method, the best smoothing factor can be obtained.

PSO is an intelligent algorithm inspired by the cooperative behavior of birds searching for food. Compared with the classical genetic algorithm, it can converge to the optimal solution faster. Assuming that the solution to the objective optimization problem is a *d*-dimensional vector and each particle in the swarm represents a possible solution, each possible solution can be evaluated by calculating the fitness of each particle in the swarm. This allows the identification of a particle *L*_best_ that contains the optimal solution in the *d*-dimensional space. Thus, the problem of determining the smoothing factor can be transformed into finding *L*_best_ using the PSO algorithm.

Assuming that *N* particles are randomly scattered in *d*-dimensional space and their positions are *L_i_* = (*l*_b1_, *l*_b2_, ···, *l*_bd_) (*i* =1, 2, ···, *n*), *L_i_* is substituted into the function *F*(*L*) to calculate its fitness *F_i_*. Based on the fitness values, the optimal positions of the *i*th particle and all particles in the swarm are obtained, denoted as *P_i_*_d_ = (*P_i_*_1_, *P_i_*_2_, ···, *P_i_*_d_) and *G_b_* = (*P*_g1_, *P*_g2_, ···, *P*_gd_) (*g* = 1, 2, ···, *N*), respectively. In this way, each particle updates its individual position based on the current position *L_i_*, the optimal position *P_i_*_d_ found by itself, and the optimal position *P*_gd_ found in the whole particle swarm.

In the initial operation, a large flight speed is necessary for the PSO algorithm to avoid being trapped in a local optimal solution. However, when the particle swarm approaches the optimal solution, the particle flying speed should not be too large as the number of iterations increases. Otherwise, it will be difficult for the particles to accurately converge on the optimal solution. To reduce fluctuations near the optimal solution, it is necessary to introduce an adaptive inertia factor *w*. As the number of iterations increases, the adaptive inertia factor *w* gradually decreases, which means that the particle flight speed decreases—ensuring the optimal smoothing factor and ultimately yielding more accurate prediction results. These relationships are described by Equations 7–9.


(7) $$v_{id}^{\left(k+1\right)}=wv_{id}^{\left(k\right)}+c_{1}r_{1}\left(p_{id}^{\left(k\right)}-l_{id}^{\left(k\right)}\right)+c_{2}r_{2}\left(p_{gd}^{\left(k\right)}-l_{id}^{\left(k\right)}\right)$$

(8) $$l_{id}^{\left(k+1\right)}=l_{id}^{\left(k\right)}+v_{id}^{\left(k+1\right)}$$

(9) $$w=W_{\text{max}}-\left(W_{\text{max}}-W_{\text{min}}\right)+\tan\left(\frac{I}{I_{\text{max}}}\right)\times \left(\frac{\pi }{4}\right)$$

Where $v_{id}^{\left(k\right)}$ is the velocity vector of particle *i* when the *k*th particle swarm is searching for the “food”; *C*_1_ and *C*_2_ are learning factors; *r*_1_ and *r*_2_ are random numbers in the (0,1) interval; and *w* is the inertia factor. *W*_max_ and *W*_min_ are the upper and lower bounds of the inertia factor, respectively. *I* and *I*_max_ are the current number and maximum iteration numbers, respectively. Once the model reaches the iteration criterion, the optimal solution is obtained.

## 4 Results and discussion

### 4.1 General characteristics

#### 4.1.1 Clinical features

There are 63 patients, including 29 men and 34 women. The median age is approximately 67 years (21~89), with 44 (69.8%) patients over 60 years old. There are 18 cases (28.6%) of Ann Arbor stages I~II and 45 cases (71.4%) at stages III~IV; 16 cases (25.4%) presented with B symptoms; 29 cases (46%) had an ECOG score of ≥ 2; 24 cases (38%) had extranodal involvement at more than two sites; 12 cases (19%) had large masses (> 7.5 cm); 31 cases (49.2%) had β_2_-MG values exceeding the normal upper limit; 30 cases (47.6%) had elevated lactate dehydrogenase (LDH) values (or levels); 50 cases (79.4%) had serum albumin values< 40 g/L; 0 case had ALT values above the normal upper limit; five cases (7.9%) had elevated AST values; and two cases (3.2%) had elevated ALP values (exceeding the normal upper limit). A total of 53 patients (84.1%) received rituximab combined with chemotherapy, while 10 cases (15.9%) received chemotherapy alone.

**Figure 3:**
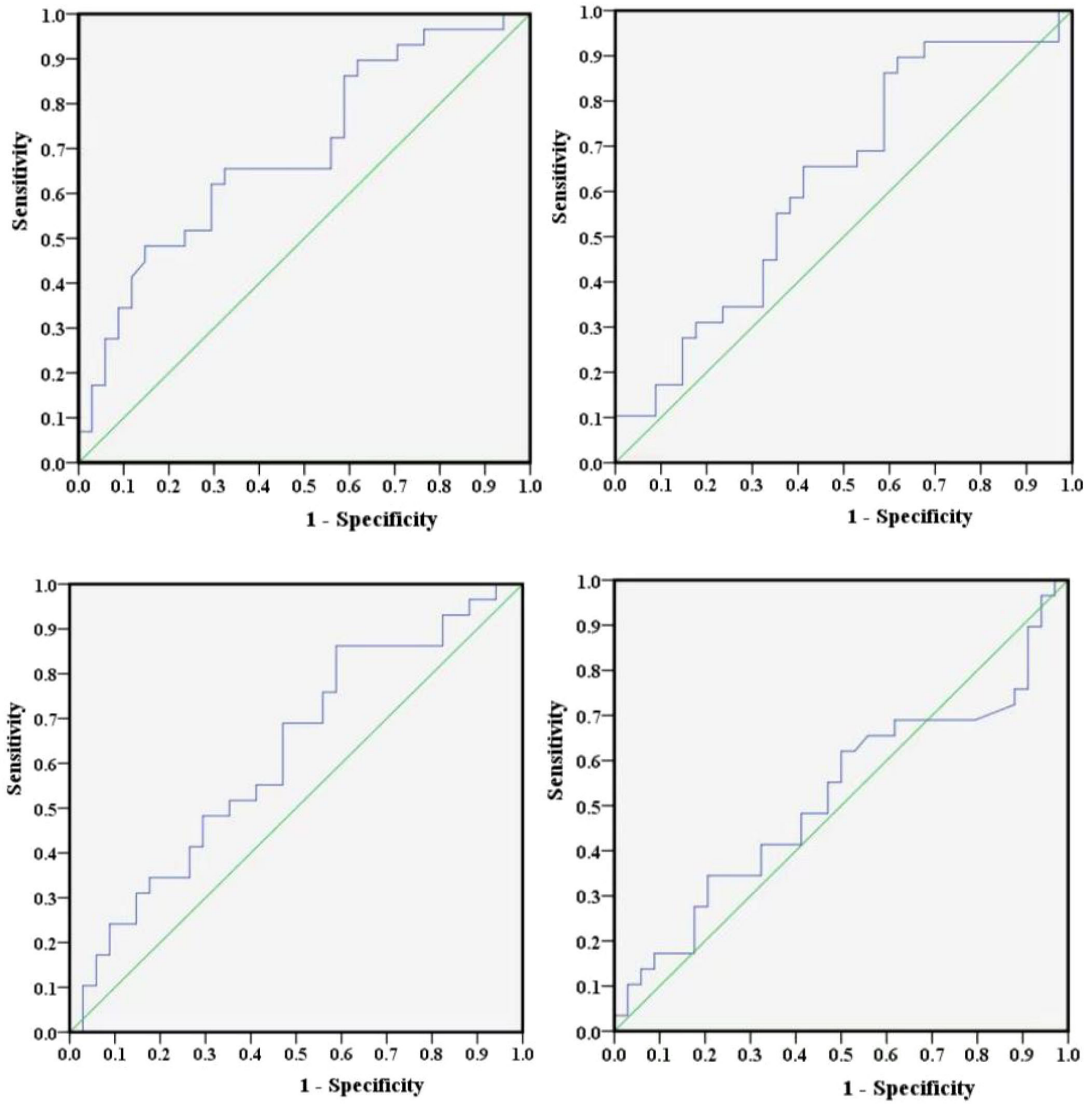
ROC curves of NLR, PLR, LMR, and SII.

In addition, this study employed Youden’s index to determine the optimal cut-off values for NLR, PLR, LMR, and SII. Youden’s index serves as a diagnostic performance metric for evaluating the ability of a screening method to distinguish between true patients and nonpatients. It is computed by subtracting (1 − specificity) from sensitivity, and the test variable value corresponding to its maximum represents the diagnostic threshold for that particular method. Furthermore, ROC curves for NLR, PLR, LMR, and SII were plotted separately using the occurrence of POD12 as the study endpoint (**Figure 3**). The sensitivity and specificity values corresponding to the maximum Youden’s index for these curves were (0.483, 0.853), (0.897, 0.382), (0.862, 0.412), and (0.345, 0.794), respectively. By integrating the actual clinical values from 63 patients, the optimal cut-off values were further determined to be 1.73, 306.494, 4.867, and 304.2, respectively.

#### 4.1.2 Pathological characteristics

Among 63 patients, 21 (33.3%) cases had pathological GCB and 42 (66.7%) cases had non-GCB; 26 cases (41.3%) were Myc positive (grades 3–4); 47 cases (69.3%) were Bcl-2 positive (≥ 50%); and 45 cases (71.4%) were Bcl-6 positive (≥ 50%). There were 12 cases (19%) of P53 expression with grades 1~2 and 51 cases (81%) with grades 3~4. Based on FISH testing, three cases (4.8%) were Myc positive, 0 cases (0%) were Bcl-2 positive, and six cases (9.5%) were Bcl-6 positive.

### 4.2 POD12 and its relationship with prognosis in patients with DLBCL

Prior to conducting the analysis, this study employed a single-proportion power test to calculate the statistical power for POD12. The sample size was set at 63 (the number of cases used), and the comparison proportion was set at 46% (the proportion of POD12 occurrence). The calculated power value was 0.927878, indicating that the sample size was sufficient for detecting statistical associations.

**Figure 4:**
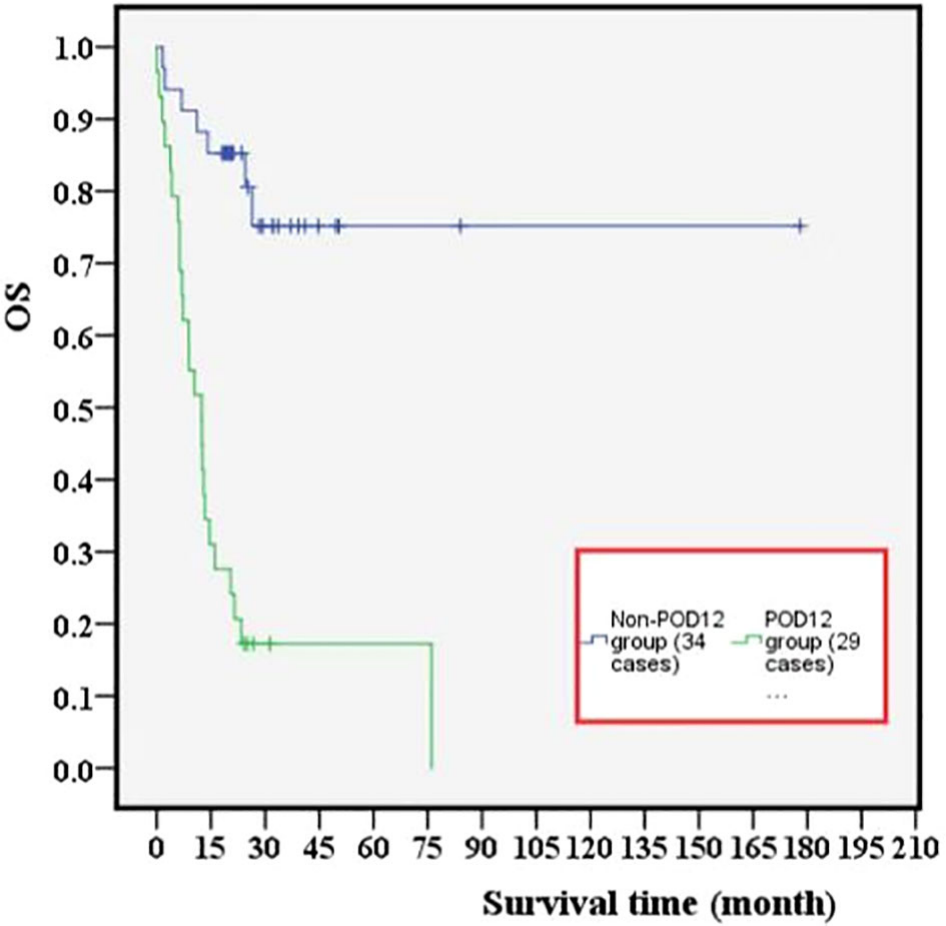
Effect of POD12 on OS in patients with DLBCL.

Among 63 previously untreated DLBCL patients, 29 cases (46%) experienced POD12. The OS values in the POD12 group were significantly lower than those in the non-POD12 group (*p*< 0.001; **Figure 4**), with 3-year OS rates of 3.45% and 23.53%, respectively.

From one-way ANOVA, POD12 is significantly correlated with progression-free survival (PFS; *p*< 0.001; **Table 1**) and OS (*p* = 0.008; **Table 2**). When factors with *p*< 0.2 in the one-way ANOVA are included in the multivariate Cox regression analysis, and the effect of first-line treatment on the prognosis of DLBCL is corrected for, POD12 remains the most significant independent prognostic factor in PFS (HR = 0.235, 95% confidence interval (CI): 0.106~0.524, *p*< 0.001; **Table 1**) and OS (HR = 0.274, 95% CI: 0.123~0.606, *p* = 0.001; **Table 2**).

**Table 1:** Univariate and multivariate Cox analysis of prognostic factors for DLBCL (PFS).

Prognostic factors	Adverse factors	One-way ANOVA		Multivariate Cox analysis	
		eta	*p-*value	HR (95% CI)	*p-*value
Sexual distinction	Male	0.02	0.878		
Age	≥ 60	0.014	0.912		
Position	Extranodal	0.133	0.3		
Origin	Non-GCB	0.014	0.913		
MYC	≥ 40%	0.035	0.787		
BCL-2	≥ 50%	0.206	0.106	1.238 (0.569–2.693)	0.59
BCL-6	≥ 30%	0.146	0.252		
P53	≥ 50%	0.009	0.942		
FMYC	+	0.052	0.683		
FBCL6	+	0.145	0.256		
Ki-67	≥ 80%	0.015	0.904		
LDH (IU/L)	≥ 250	0.353	0.004	1.806 (0.869–3.751)	0.113
Seralbumin (g/L)	≥ 40	0.024	0.855		
AST	≥ 50	0.043	0.739		
β_2_-MG	≥ 3	0.138	0.279		
Stage	III, IV	0.278	0.027	2.534 (0.987–6.507)	0.053
B symptom	Yes	0.117	0.362		
Extranodal number	≥ 2	0.159	0.214		
Bulky	Yes	0.004	0.974		
ECOG	≥ 2	0.235	0.063	0.823 (0.404–1.676)	0.592
*R* ^2^	No	0.015	0.910		
POD12	Yes	0.435	< 0.001	0.235 (0.106–0.524)	< 0.001
NLR	≥ 1.73	0.194	0.127	0.813 (0.309–2.138)	0.675
PLR	≥ 306.494	0.062	0.629		
SII	≥ 304.2	0.182	0.153	0.675 (0.275–1.657)	0.391
LMR	≥ 4.867	0.008	0.948		

**Table 2:** Univariate and multivariate Cox analysis of prognostic factors in DLBCL (OS).

Prognostic factors	Adverse factors	One-way ANOVA		Multivariate Cox analysis	
		eta	*p*-value	HR (95% CI)	*p*-value
Sexual distinction	Male	0.076	0.555		
Age	≥ 60	0.013	0.919		
Position	Extranodal	0.08	0.533		
Origin	Non-GCB	0.015	0.906		
MYC	≥ 40%	0.038	0.768		
BCL-2	≥ 50%	0.193	0.129	1.17 (0.535–2.555)	0.694
BCL-6	≥ 30%	0.154	0.227		
P53	≥ 50%	0.028	0.826		
FMYC	+	0.058	0.649		
FBCL6	+	0.139	0.279		
Ki-67	≥ 80%	0.041	0.750		
LDH(IU/L)	≥ 250	0.358	0.004	2.542 (1.219–5.303)	0.013
Seralbumin (g/L)	≥ 40	0.028	0.827		
AST	≥ 50	0.064	0.616		
β_2_-MG	≥ 3	0.101	0.431		
Stage	III, IV	0.234	0.065	1.688 (0.662–4.307)	0.273
B symptom	Yes	0.143	0.263		
Extranodal number	≥ 2	0.195	0.126	0.713 (0.337–1.507)	0.376
Bulky	Yes	0.032	0.806		
ECOG	≥ 2	0.211	0.097	0.692 (0.344–1.393)	0.302
*R* ^2^	No	0.068	0.596		
POD12	Yes	0.334	0.008	0.274 (0.123–0.606)	0.001
NLR	≥ 1.73	0.185	0.148	1.185 (0.391–3.585)	0.764
PLR	≥ 306.494	0.051	0.690		
SII	≥ 304.2	0.164	0.198	0.525 (0.182–1.518)	0.234
LMR	≥4.867	0.043	0.736		

### 4.3 Risk factor analysis of POD12

Among the 63 patients who underwent immunohistochemical detection, univariate logistic regression analysis—adjusted for the first-line chemotherapy regimen—identified LDH ≥ 250 IU/L, β_2_-MG ≥ 3 µmol/L, stages III–IV, ECOG ≥ 2, NLR ≥ 1.73, SII ≥ 304.2 as potential risk factors for POD12 (**Table 3**). Furthermore, multivariate logistic regression analysis revealed that β_2_-MG ≥ 3 µmol/L and ECOG ≥ 2 were independent risk factors for POD12 (*p*< 0.05; **Table 4**).

**Table 3:** Univariate logistic regression analysis adjusted for the first-line chemotherapy regimen for POD12.

Prognostic factors	Adverse factors	*β*	SE	Wald	OR	95% CI		*p*-value
Sexual distinction	Male	0.61	0.514	1.408	1.841	0.672	5.045	0.235
Age	≥ 60	0.539	0.563	0.916	1.714	0.569	5.169	0.338
Position	Extranodal	0.466	0.51	0.835	1.594	0.586	4.331	0.361
Origin	Non-GCB	0.486	0.545	0.793	1.625	0.558	4.73	0.373
MYC	≥ 40%	0.666	0.559	1.416	1.946	0.65	5.823	0.234
BCL-2	≥ 50%	0.234	0.542	0.185	1.263	0.436	3.658	0.667
BCL-6	≥ 30%	0.205	0.607	0.114	1.227	0.374	4.031	0.736
P53	≥ 50%	0.008	0.513	0	1.008	0.369	2.758	0.987
FMYC	+	0.56	1.252	0.2	1.75	0.15	20.35	0.655
FBCL6	+	1.337	1.184	1.275	3.808	0.374	38.777	0.259
Ki-67	≥ 80%	0.87	0.687	1.604	2.386	0.621	9.169	0.205
LDH (IU/L)	≥ 250	1.379	0.536	6.63	3.973	1.39	11.353	0.01
Seralbumin (g/L)	≥ 40	0.811	0.664	1.491	2.25	0.612	8.27	0.222
AST	≥ 50	0.613	0.95	0.416	1.846	0.287	11.889	0.519
β_2_-MG	≥ 3	2.167	0.583	13.832	8.73	2.787	27.348	< 0.001
Stage	III, IV	1.089	0.605	3.239	2.971	0.908	9.729	0.072
B symptom	Yes	0.468	0.593	0.623	1.597	0.499	5.108	0.43
Extranodal number	≥ 2	0.806	0.529	2.325	2.24	0.794	6.316	0.127
Bulky	Yes	1.138	0.723	2.476	3.12	0.756	12.873	0.116
ECOG	≥ 2	1.517	0.542	7.823	4.56	1.575	13.205	0.005
*R* ^2^	No	0.189	0.69	0.075	1.208	0.312	4.672	0.784
NLR	≥ 1.73	1.284	0.717	3.211	3.611	0.887	14.709	0.073
PLR	≥ 306.494	0.742	0.603	1.514	2.1	0.644	6.846	0.218
SII	≥ 304.2	1.095	0.651	2.825	2.989	0.834	10.717	0.093
LMR	≥ 4.867	0.028	0.666	0.002	1.029	0.279	3.797	0.966

**Table 4:** Multivariate logistic regression analysis for POD12.

Prognostic factors	Adverse factors	*β*	SE	Wald	OR	95%CI		*p*-value
LDH (IU/L)	≥ 250	0.251	0.784	0.103	0.778	0.167	3.615	0.749
β_2_-MG	≥ 3	2.615	0.872	9.002	13.671	2.476	75.468	0.003
Stage	III, IV	0.991	0.87	1.295	2.693	0.489	14.831	0.255
ECOG	≥ 2	2.299	0.804	8.178	9.961	2.061	48.14	0.004
NLR	≥ 1.73	0.408	1.465	0.077	1.503	0.085	26.569	0.781
SII	≥ 304.2	1.371	1.348	1.035	3.94	0.281	55.323	0.309

**Figure 5:**
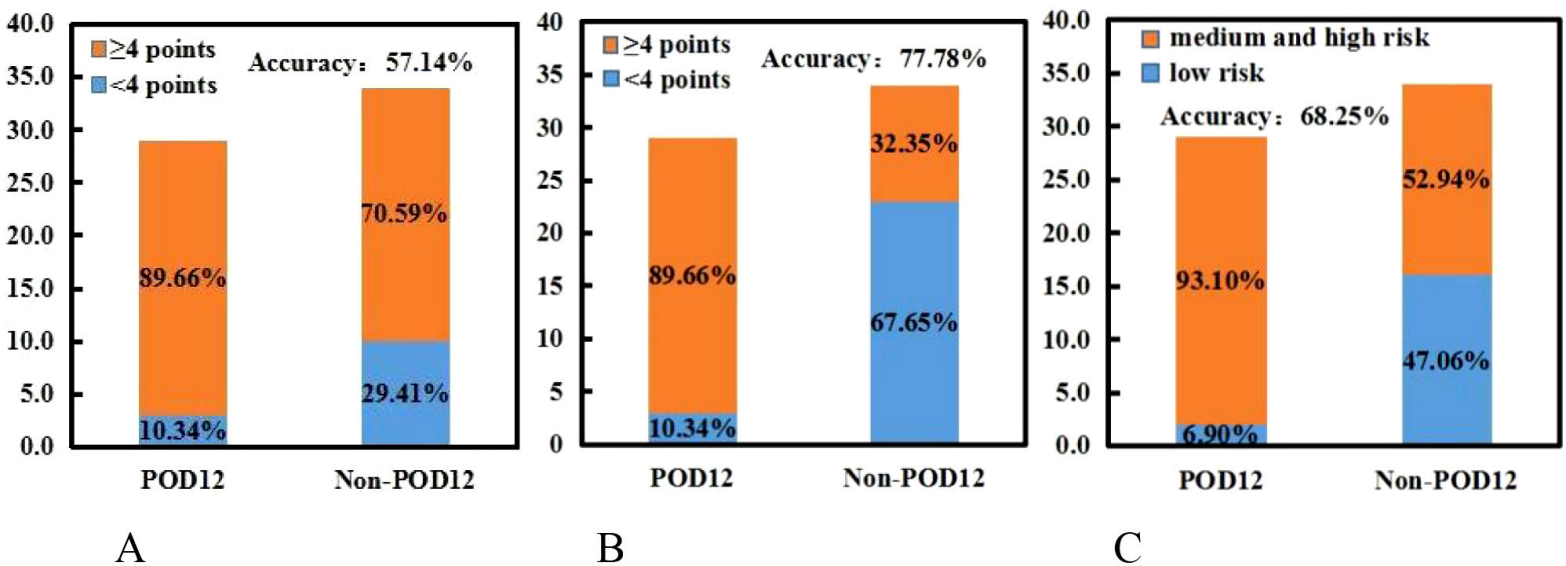
NCCN-IPI and two prediction models, **(A, B)**. **(A)** NCCN-IPI. **(B)** Prediction model **(A)**. **(C)** Prediction model **(B)**.

According to the analysis of POD12 in previously untreated DLBCL patients, the risk factors for POD12 are LDH ≥ 250 IU/L, β_2_-MG ≥ 3 µmol/L, stages III–IV, ECOG ≥ 2 score, NLR ≥ 1.73, and SII ≥ 304.2. From the prediction model A, which includes the above six factors, the logistic regression coefficient *β* values are 0.251, 2.615, 0.991, 2.299, 0.408, and 1.371, respectively. The larger the regression coefficient, the stronger the correlation between the corresponding risk factors and POD12. The regression coefficient value involving β_2_-MG ≥ 3 µmol/L is the largest, giving a score of 2, while the risk factors LDH ≥ 250 IU/L, stages III–IV, ECOG ≥ 2, NLR ≥ 1.73, and SII ≥ 304.2 are each assigned 1 point. The ROC curve was obtained based on the patient’s scores and whether POD12 occurred. The area from the curve is 0.846 (95% CI: 0.749–0.944), and the best cut-off value is a score of 4, which divides the patients into a low-risk group (< 4) and a high-risk group (≥ 4). Upon verification in 63 patients with DLBCL with complete clinical data, it was found that 26 patients with previously untreated DLBCL had scores less than 4, among whom 23 (88.46%) did not develop POD12. POD12 occurred (i.e., ≥ 4 score) in 26 of 37 patients (70.27%). The positive predictive rate (PPV) is 70.27%, the negative predictive rate (NPV) is 88.46%, and the overall accuracy is 77.78%. The platelet-to-lymphocyte ratio (PLR) is 2.769, indicating that the risk of POD12 in patients with DLBCL with a score of ≥ 4 was 2.769 times higher than in those with a score of< 4. Model A can also be used to predict the OS of patients. For patients with a score of< 4, the median OS was not reached, whereas the median OS for those with a score of ≥ 4 was 13 months; the difference was statistically significant (*p*< 0.001). Logistic regression analysis corrected by the first-line treatment regimen showed that the risk of POD12 in patients with scores ≥ 4 was significantly higher than in those with scores< 4 (OR = 18.121, 95% CI: 4.494–73.078, *p*< 0.001; **Table 5**). Compared with NCCN-IPI, the accuracy of POD12 prediction increased from 57.14% to 77.0%, and the risk of POD12 in the high-risk patients was significantly higher than in low-risk patients (OR = 12, 95% CI: 2.456–58.631, *p* = 078%; **Figure 5**). In addition, prediction model B was established based on the independent risk factors β_2_-MG and ECOG for POD12. The high-risk group was defined as having ECOG ≥ 2 and β_2_-MG ≥ 3 µmol/L; the medium-risk group included patients with either ECOG ≥ 2 and β_2_-MG< 3 µmol/L, or ECOG< 2 and β_2_-MG ≥ 3 µmol/L; and the low-risk group included those with ECOG< 2 and β_2_-MG< 3 µmol/L. According to the logistic regression analysis corrected by the first-line treatment regimen, the predictive ability of this model was medium-high at 002 (see **Table 5**). Its prediction accuracy was higher than that of NCCN-IPI but lower than that of prediction model A (see **Figure 5**). Therefore, in this study, prediction model A was selected as the final prediction model for POD12.

**Table 5:** Comparison of single-factor logistic regression analysis between the NCCN-IPI and the prediction model.

Model	Adverse factors	*β*	SE	Wald	OR	95% CI		*p*-value
NCCN-IPI	≥ 4	1.284	0.717	3.211	3.611	0.887	14.709	0.073
Model A	≥ 4	2.897	0.711	16.581	18.121	4.494	73.078	< 0.001
Model B	Medium and high risk	2.485	0.809	9.426	12	2.456	58.631	0.002

Different from the PFS or OS, POD12 is not influenced by deaths caused by other factors (such as treatment-related adverse reactions) or by second-line treatment. It may better reflect the invasiveness and/or treatment resistance of the disease itself. At present, some conventional clinical prognostic evaluation systems, such as the international prognostic index (IPI) (10), cell-of-origin classification (11, 12), simultaneous Bcl-2 and C-Myc and/or Bcl-6 rearrangement, dual expression (DE) of C-Myc and Bcl-2, increased expression of mutant p53 protein (13–16), high-grade B-cell lymphoma, double-hit/triple-hit DLBCL (15, 17, 18), can be used to predict the overall survival of patients with DLBCL. However, the relationship between these prognostic factors and POD12 in DLBCL remains uncertain (19). In addition, patients with POD12 may initially achieve CR with first-line treatment but then experience rapid disease progression. Their tumor cells may not respond well to follow-up therapies, resulting in poor overall survival (OS) (20, 21). Similarly, POD12 has been shown to have important prognostic value. Ma et al. (4) found that DLBCL patients treated with first-line rituximab + cyclophosphamide + adriamycin + vincristine + prednisone (R-CHOP) had significantly lower OS rates if they experienced POD12 compared to those who did not (*p*< 0.001). These patients also responded poorly to subsequent rescue treatment, indicating that POD12 carries strong prognostic significance across various conventional treatment regimens.

At present, there are few investigations on the predictive factors of POD12, with high heterogeneity in biological characteristics. It is necessary to establish a POD12 prediction model to help identify high-risk groups, formulate personalized therapy plans, and improve prognosis. In this study, the incidence of POD12 is high (46%), and OS is significantly worse in patients with early progression compared to those without. This may be closely related to factors such as advanced disease stage, high tumor burden, and limited sample size, which need to be further verified in large-sample clinical trials. In addition, this study included patients who received immunochemotherapy and chemotherapy, and the effect of first-line treatment on the prognosis of DLBCL was corrected; POD12 remained the most significant independent prognostic factor for both PFS and OS. This work confirms that POD12 is a reliable adverse prognostic factor for DLBCL. Therefore, it is necessary to identify high-risk patients early and implement individualized treatment in a timely manner to reduce the incidence of POD12. Ma et al. (4) found that, in addition to the progression of the disease, neither the prognostic factors in traditional IPI nor others, such as double expression, TP53, and COO, can serve as independent predictors of POD12. Combining the CD79B mutation, PIM1 mutation, and Ann Arbor staging can further improve prediction efficiency, with POD12 showing the highest accuracy. Therefore, some scholars have attempted to predict POD12 by combining clinical features and gene mutations. However, these prediction tools require expensive and time-consuming gene sequencing and related tests, which are currently difficult to implement in clinical practice. After screening the baseline clinical and pathological factors, we found that certain risk factors, such as LDH ≥ 250 IU/L, β_2_-MG ≥ 3 µmol/L, stages III–IV, ECOG ≥ 2 score, NLR ≥ 1.73, and SII ≥ 304.2, are strongly associated with POD12. Accordingly, a new prognostic model was established. Sixty-three previously untreated DLBCL patients with complete clinical data were divided into low-risk (score 0–3) and high-risk (score ≥ 4) groups. The risk of the POD12 in previously untreated DLBCL patients with a score ≥ 4 was 2.769 times higher than that in patients with a score of 0–3. In addition, the POD12 prediction model can also predict the OS of previously untreated DLBCL patients.

There is currently no optimal prognostic model for diffuse large B-cell lymphoma that accurately predicts early disease progression. Clinical indicators, immune microenvironment, and pathological indicators may influence the prognosis of DLBCL. The indicators selected in this study provide a comprehensive evaluation by combining clinical, pathological, and microenvironment-related indicators. It is well known that inflammation plays an important role in tumor progression and treatment response, and peripheral blood cell counts, which to some extent reflect the inflammatory state, are closely related to the progression of cancer (22, 23). In this work, prognostic indicators of inflammation are included. NLR (24–28) and SII (29) are considered to have prognostic value for DLBCL. A recent meta-analysis showed that NLR was associated with poor OS and was considered a low-cost prognostic factor in 2,297 patients with DLBCL (30). Based on neutrophils, platelets, and lymphocytes, SII may reflect inflammatory status and tumor activity more accurately than PLR and NLR, and it has been proven to be an independent predictor of various malignancies, such as breast cancer and lung cancer (31, 32). However, the data on non-Hodgkin lymphoma are limited. To our knowledge, the relationship between the above indicators and POD12 outcomes in DLBCL has not been explored. Therefore, we conducted this investigation to evaluate the prognostic value of NLR, PLR, SII, and LMR in POD12 of DLBCL and found that NLR and SII were prognostic risk factors for POD12. In addition, we found that patients with LDH ≥ 250 IU/L and stages III–IV were more likely to develop POD12, but these were not independent risk factors. Myc, p53, Bcl-2, and Bcl-6 did not influence the occurrence of POD12.

Among the clinical factors included in this study, β_2_-MG and ECOG scores were identified as the independent risk factors for POD12, while LDH ≥ 250 IU/L, stages III–IV, NLR ≥ 1.73, and SII ≥ 304.2 were also associated with increased risk. In diagnosis, attention should be paid to the identification of these high-risk patients, and personalized therapy should be initiated promptly to improve prognosis. In the new model developed in this study, patients were divided into high- and low-risk groups, with the former showing a significantly higher risk of POD12. Compared with NCCN-IPI alone, the new model demonstrated improved sensitivity and accuracy in predicting POD12, accurately identifying 70.27% of POD12 cases, though with slightly reduced specificity. Given the current lack of efficient POD12 prediction models, and considering that this new model is more economical and simpler than molecular clinical models, the findings of this study hold practical significance.

### 4.4 Risk level prediction for POD12 with CNN-LSTM

For the deep learning prediction model of POD12 using CNN-LSTM and PSO-GRNN, as mentioned above, LDH, β2-MG, III-IV, ECOG, NLR, and SII are selected as the risk factors for POD12. These characteristic variables are divided into two combinations: one consists of LDH, β2-MG, and stages III–IV, and the other consists of ECOG, NLR, and SII.

In the pretreatment process, a small number of missing monitoring results are filled using the “before and after mean” method (i.e., *x_i_* = (*x_i −_*_1_ + *x_i_*_+ 1_)/2). A control parameter *μ* (*μ* = 0.85) is introduced to correct them with *x_i_* = *μx_i_* (*x_i_* = LDH, β2-MG, III-IV, ECOG, NLR, and SII), to ensure the relative stability of the monitoring time series and the reliability of the prediction results.

The risk rank of POD12 can be evaluated using a comprehensive index, which is calculated from each single index of the risk rank reflected by each POD12 characteristic variable at each time. The calculation of the POD12 comprehensive risk index mainly includes the following steps.

Calculation of the risk factors of a single index (*W_i_* (*t*)) (Equation 10)


(10) $$W_{i}(t)=\frac{\left|\left|x(t)\right|-\bar{x_{0}}\right|}{x_{\max}-\bar{x_{0}}}$$

Where x(t) is the amplitude of the monitoring index (such as LDH, β2-MG, III-IV, ECOG, NLR, and SII) at time *t*, and $\bar{x_{0}}$ and $x_{\max}$ are the average and maximum values of the amplitude of the monitoring index under the normal conditions.

Determining the weight factor (*p_i_* (*t*)) of the risk factors of the single index to calculate the comprehensive risk factors (*W_z_* (*t*)) of the multivariable characteristics of POD12, based on the risk factors and their respective weights (Equation 11).


(11) $$W_{z}(t)=\sum_{i=1}^{n}W_{i}(t)\cdot p_{i}(t)$$

The rank of the risk for POD12 could then be determined by the comprehensive risk index. The risk ranking is divided into four categories: risk, subsidiary risk, subsidiary safe, and safe.

Based on chaos theory, for six characteristic variables of POD12 (i.e., LDH, β2-MG, stages III–IV, ECOG, NLR, and SII), the delay time (*τ*) was calculated using the mutual information method, and the embedding dimension (*m*) was determined using the false nearest-neighbor method, where *m* corresponds to the value with the minimum false proximity rate.

The characteristic time series were predicted using a single-layer structure in LSTM. The number of neurons is set to 100, and the hyperparameter *λ* is 0.01. The CNN-LSTM model was used for training. In the process of training the model, the training time step was set to the embedded dimension *m*, the activation function was Relu, and the optimizer was Adam. The number of epochs was set to 100, and the learning rate was 0.01, with an experimental time step of 1 h.To assess the performance of the prediction models, the mean absolute percentage error (MAPE) is a commonly used criterion. Specifically, given a dataset $y=\{y_{1},y_{2},\ldots ,y_{N}\}$ , let the predicted values output by a certain prediction model be denoted as

$\overset{\wedge }{y}=\{\overset{\wedge }{y_{1}},\overset{\wedge }{y_{2}},\ldots ,\overset{\wedge }{y_{N}}\}$ , then Equation 12


(12) $$\text{MAPE}=\frac{1}{N}\sum_{k=1}^{N}\left|\frac{y_{k}-\overset{\wedge }{y_{k}}}{y_{k}}\right|\times 100\%$$

It can be seen from the calculation formula that the smaller the MAPE value, the better the performance of the model. In order to better describe the prediction ability of the model, this study cited the evaluation scale introduced in Xu et al. (33). Its details are as follows: when the MAPE is less than 10%, the prediction ability is considered “highly accurate”; when the MAPE is between 10% and 20%, the prediction ability is “good”; when the MAPE is between 20% and 50%, the prediction ability is “reasonable”; and when the MAPE is greater than 50%, the prediction ability is considered “weak and inaccurate”.

In addition, the mean absolute scaled error (MASE) can also be used to measure the performance of prediction models and is expressed as Equation 13:


(13) $$\text{MASE}=\frac{1}{N}\sum_{k=1}^{N}\frac{\left|y_{k}-\overset{\wedge }{y_{k}}\right|}{\frac{1}{N}\sum_{k=1}^{N}\left|y_{k}-\bar{y}\right|}$$

Subsequently, to comparatively evaluate the predictive performance of the models, this study divided the case dataset into a training set and a test set at a ratio of 9:1. First, the GRNN, DB-LSTM [introduced by Zhou and Xu (34)], DeepSurv [introduced by Katzman et al. (35)], and CNN-LSTM models were trained on the training set. After training, the models were applied to the test set, with the results presented in **Table 6**.

**Table 6:** MAPE (%) and MASE values of the different prediction models.

Evaluation indicators	DB-LSTM	GRNN	DeepSurv	CNN-LSTM
MAPE	20.873	23.612	15.385	9.739
MASE	3.036	3.377	2.354	1.348

For the prediction of the POD12 comprehensive risk index using LDH, β_2_-MG, III-IV, ECOG, NLR, and SII, as shown in **Table 6**, CNN-LSTM exhibits the smallest MAPE and MASE values (bold values in **Table 6**), indicating highly accurate prediction performance. Compared with the maximum value of MAPE among the four models, the MAPE value of CNN-LSTM is reduced by 58.75%. In addition, the MASE values of the other three models are 2.25 times, 2.51 times, and 1.75 times that of CNN-LSTM, respectively.

### 4.5 Assessment of the impact of different combinations on the risk level of POD12

To verify the effectiveness of the PSO-GRNN method, experiments were conducted on the discrimination of the risk level of POD12 and the prediction of the risk level of POD12 in the future.

According to the characteristic variables of two combinations (“LDH, β2-MG, and III-IV” and “ECOG, NLR, and SII”), the POD12 risk levels were comprehensively evaluated.

In order to obtain the optimal model, the fourfold cross-validation method is adopted. The ratio of the training set to the test set is 9:1. The number of particles is 10, the learning factor is 0.2, and the inertia weight is 0.7. The iteration stop condition is that the number of cycle steps exceeds 100 or the training error is less than 0.0010. Taking the obtained weight threshold as the initial value, the network is trained using the GRNN algorithm.

**Figure 6:**
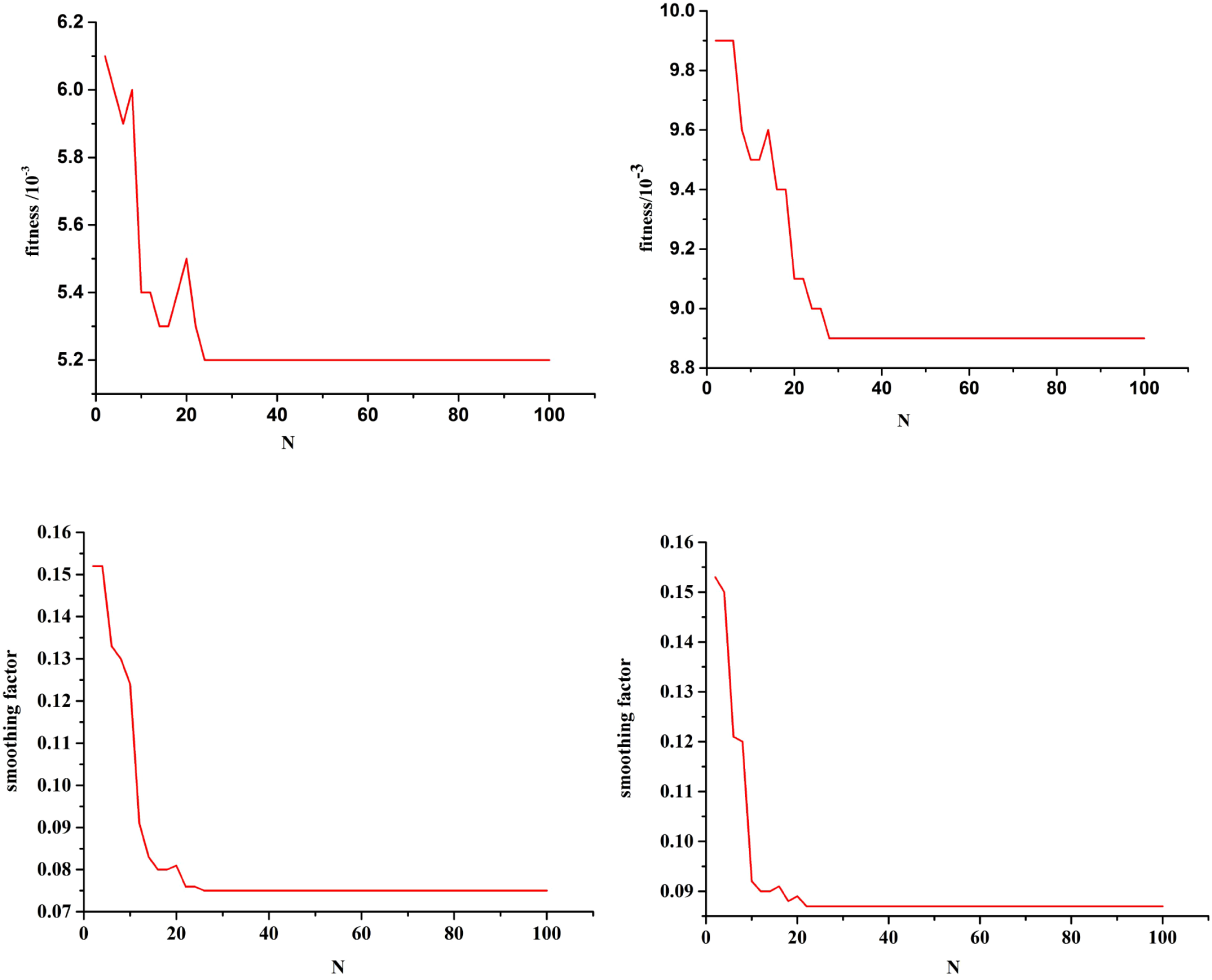
Fitness function optimization curve and smoothing optimization curve. Combination (LDH, β2-MG, and III-IV) Combination (ECOG, NLR, and SII). Combination (LDH, β2-MG, and III-IV) Combination (ECOG, NLR, and SII).

**Figure 6** shows the optimization curves of the fitness function and the smoothing factor. Single-step prediction was adopted, and the mean square error between the predicted and test values is used as the fitness. The smaller the fitness, the better the training effect of the model. Based on the characteristic variables of two combinations (“LDH, β2-MG, and III-IV” and “ECOG, NLR, and SII”), the optimal fitness values are 0.0052 and 0.0089, and the optimal smoothing factors are 0.0753 and 0.0868, respectively. The improved PSO algorithm can effectively avoid missing the optimal solution and can find the global optimal solution. Using the optimized GRNN, the risk level of the current state of POD12 is determined.

To predict the future risk level of POD12, the CNN-LSTM model was first used to forecast the future state values of the characteristic variables. Subsequently, the PSO-GRNN model was employed to predict the future state value of the characteristic variables (i.e., the risk level of POD12 at the time of *t* + 1). To maintain methodological consistency, the training and verification sets were kept unchanged. However, the prediction dataset consisted of the forecasted future state values of the characteristic variables associated with POD12. Fourfold cross-verification was adopted, with parameter settings remaining consistent throughout the process.

**Figure 7:**
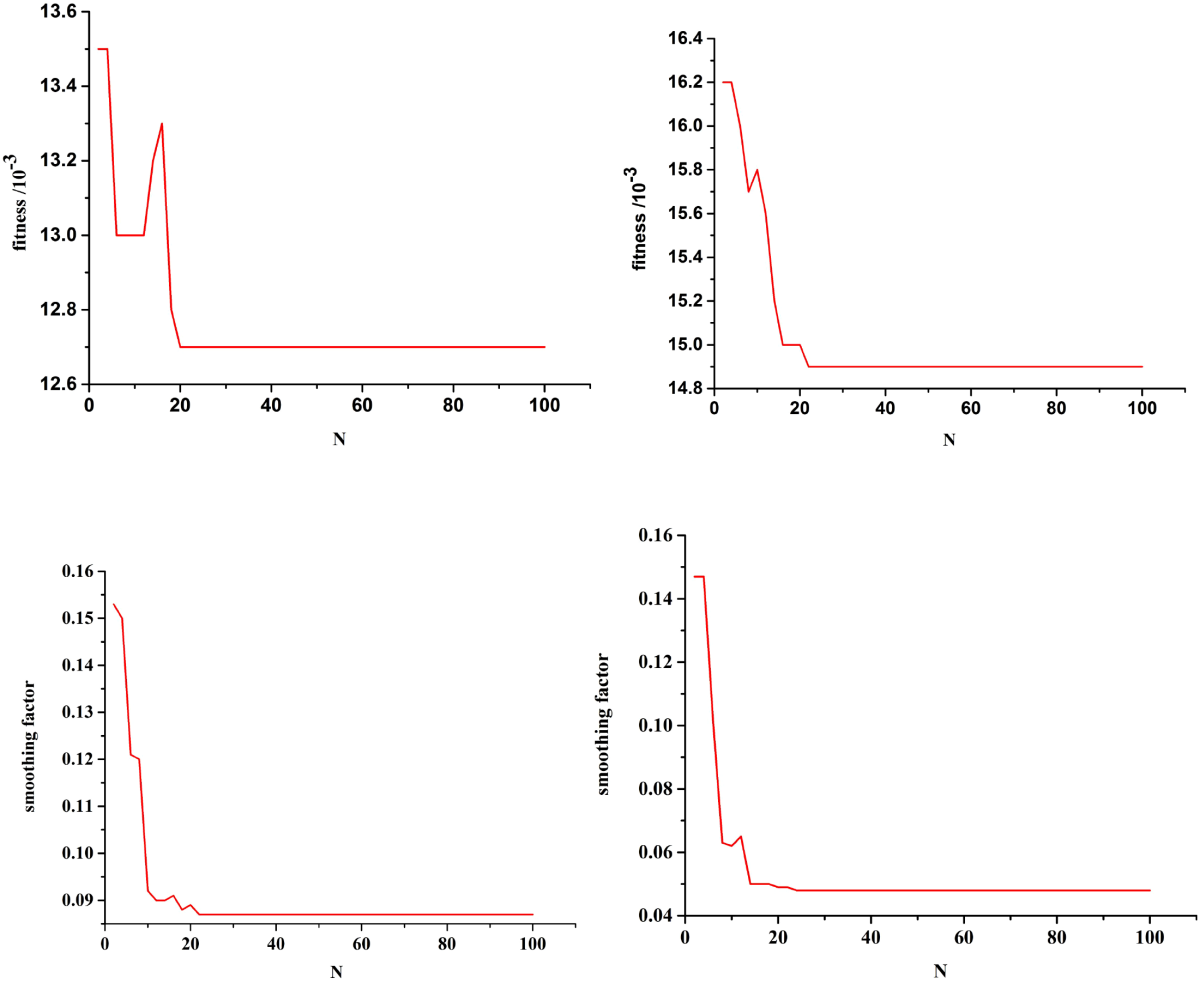
Fitness function optimization curve with future state of POD12, and smoothing optimization curve with future state of POD12. Combination (LDH, β2-MG, and III-IV) Combination(ECOG, NLR, and SII). Combination (LDH, β2-MG, an.

Single-step prediction was adopted, and the mean square error between the predicted and test values was used as the fitness. **Figure 7** shows the optimization curves for the fitness function and the smoothing factor for future POD12 prediction. The algorithm demonstrated robustness; for example, in the combination of LDH, β2-MG, and stages III–IV, the optimal fitness value and smoothing factor were 0.0127 and 0.0859, respectively, which closely align with the previously reported experimental results.

Using the optimized GRNN, the risk level of POD12 in the future was predicted at Shanxi Bethune Hospital from January 2016 to June 2020. It can be seen that, with the help of the method in this work, not only can the POD12 evolution state be accurately fitted, but the risk level of the current state of POD12 can also be judged, and that in the future can be predicted as well. The prediction accuracy of the characteristic variable and risk level of the POD12 in the future is high. This can provide an important basis for timely mastering the future state of POD12 activities, i.e., the risk level of POD12 in the hospital.

To sum up, by analyzing the incidence and risk factors of POD12 in patients with previously untreated DLBCL, a clinical prediction model of POD12 was established, and the prediction efficiency of the model was also verified. It was further found that the model can also predict OS. Therefore, the prediction model is helpful to evaluate, simply and effectively, the risk of POD12 in previously untreated DLBCL patients, and then formulate personalized therapy plans for high-risk patients to reduce the risk of POD and prolong overall survival time. However, this study is a single-center retrospective study with a limited sample size and a lack of external validation, and it is unclear whether the POD12 prediction model based on clinical characteristics and immunohistochemical molecules is applicable to populations in different regions and various treatment cases. It is urgent to further explore large-sample clinical trials under the cooperation of multiple centers. The CNN-LSTM and PSO-GRNN models are the most suitable to predict the risk level of the POD12 in the future. This can provide an important basis for timely mastering the future state of POD12.

## 5 Conclusion

The POD12 prediction model, based on LDH, β_2_-MG, stage, ECOG, NLR, and SII, can be used to effectively predict the early recurrence and progression of DLBCL. Among the models evaluated, CNN-LSTM and PSO-GRNN demonstrated the highest suitability for forecasting the risk level of POD12, providing an important basis for the timely assessment of patient prognosis. In addition, the model can be applied clinically through the following steps to support physicians in developing optimized treatment plans:

(1) Indicator input: clinicians input six routine clinical indicators—LDH, β_2_-MG, stage, ECOG, NLR, and SII;
(2) Automatic scoring: the model calculates the POD12 risk score in real time;
(3) Risk stratification: for low-risk patients, standard R-CHOP therapy can be recommended; for high-risk patients, personalized intensive regimens may be considered in combination with other indicators;
(4) Dynamic monitoring: clinicians update patient indicators every month and use the CNN-LSTM model to predict the probability of disease progression within 12 months in a single-step prediction.

## 6 Future directions

While this study primarily employs statistical and deep learning methodologies to identify prognostic patterns in clinical data, we acknowledge that incorporating biological principles could enhance model interpretability and generalizability. Our immediate next steps include:

(1) Integrating single-cell transcriptomics to map molecular subtypes to POD12 trajectories;
(2) Modeling tumor-immune ecosystem dynamics using spatially resolved proteomics as biological constraints for CNN-LSTM;
(3) Developing mechanistic hybrid models in which neural networks parameterize differential equations describing lymphoma proliferation and immune interactions. These efforts aim to bridge data-driven predictions with causal biological reasoning.

## Data Availability

The data analyzed in this study is subject to the following licenses/restrictions: The datasets generated during and analysed during the current study are not publicly available due the data in the study involves personal privacy but are available from the corresponding author on reasonable request. Requests to access these datasets should be directed to Hui Wang, wh_data007@zufe.edu.cn.
